# Calcineurin Inhibitor-Based Triple Immunosuppressive Therapy Induces Profibrotic Changes in the Jejunal Mucosa of Male Rats: A Digital Image Analysis of Collagen Fibers

**DOI:** 10.3390/biomedicines14092064

**Published:** 2026-09-15

**Authors:** Kamila Szumilas, Aleksandra Wilk, Paweł Szumilas, Karolina Klimek, Karolina Kędzierska-Kapuza

**Affiliations:** 1 Department of Physiology, Pomeranian Medical University, 70-111 Szczecin, Poland; kamila.szumilas@pum.edu.pl; 2 Department of Histology and Embryology, Pomeranian Medical University, 70-111 Szczecin, Poland; aleksandra.wilk@pum.edu.pl; 3 Department of Social Medicine and Public Health, Pomeranian Medical University, 71-210 Szczecin, Poland; 4 Department of Economics and Health Care Management, Faculty of Public Health in Bytom, Medical University of Silesia in Katowice, 40-007 Katowice, Poland; karolina.klimek@sum.edu.pl; 5 Centre of Diabetology, National Medical Institute of the Ministry of Interior Affairs and Administration, 02-507 Warsaw, Poland; karolina.kedzierska@gmail.com

**Keywords:** immunosuppressive drugs, calcineurin inhibitors, cyclosporine A, tacrolimus, jejunum, collagen fibers, digital image analysis

## Abstract

**Background/Objectives:** Long-term immunosuppressive therapy administered to solid organ transplant recipients may induce adverse effects in various tissues, including the gastrointestinal tract. The aim of the present study was to evaluate the influence of prolonged treatment with calcineurin inhibitor (CNI)-based triple immunosuppressive regimens on the morphology of the jejunal mucosa in male rats. Particular attention was paid to fibrotic changes occurring within the mucosal connective tissue, including the intestinal villi and lamina propria. **Methods**: Animals were treated for six months with regimens containing either cyclosporine A (CyA) or tacrolimus (TAC) in combination with rapamycin and prednisone. To assess the content of collagen fibers, jejunal sections were stained using the Mallory trichrome method and subsequently subjected to quantitative digital image analysis. **Results**: Histological examination demonstrated increased collagen deposition in the jejunal mucosa of rats receiving immunosuppressive treatment compared with the control group. Quantitative analysis confirmed an increase in collagen fiber content, indicating enhanced extracellular matrix accumulation and changes consistent with fibrotic remodeling of the mucosal connective tissue. The highest collagen fiber content was observed in animals treated with the cyclosporine-based regimen. **Conclusions**: These findings suggest that long-term administration of CNI-based triple immunosuppressive therapy may be associated with changes consistent with fibrotic remodeling of the jejunal mucosa. The present study provides novel data on the effects of clinically relevant multidrug immunosuppressive protocols, whereas most previous experimental studies investigating the effects of immunosuppressive agents have focused on single-drug exposure in animal models or in vitro systems.

## 1. Introduction

Immunosuppression plays a key role in transplant medicine, where it prevents the rejection of transplanted organs, as well as in the treatment of autoimmune diseases, in which the immune system mistakenly attacks the body’s own tissues. Immunosuppressive regimens used in parenchymal organ transplantation have evolved considerably in recent years [1].

The type of immunosuppressive therapy required depends on the underlying disease. Monotherapy, involving the use of a single drug, is commonly employed in the treatment of autoimmune diseases, whereas immunosuppressive therapy in organ transplant recipients most often consists of a combination of three drugs. The choice of immunosuppressive agents depends primarily on the type of transplanted organ and the presence of comorbidities. Recipients of parenchymal organ transplants require lifelong suppression of the immune response. In clinical practice, long-term graft survival depends on the careful adjustment and monitoring of immunosuppressive regimens. These drugs play a crucial role in maintaining graft function and preventing transplant rejection [2]; however, they may also cause a variety of adverse effects [3].

Maintenance immunosuppressive therapy commonly includes calcineurin inhibitors (CNIs), antiproliferative agents, mTOR inhibitors, and corticosteroids. In selected cases, calcineurin inhibitors may be replaced with belatacept. Each class of immunosuppressive drugs targets different pathways and exhibits distinct mechanisms of action (Table 1).

**Table 1:** Main classes of immunosuppressive drugs used in maintenance therapy after solid organ transplantation and their mechanisms of action.

Calcineurin Inhibitors (CNIs)	Cyclosporine A CyA	Cyclosporine binds to cyclophilin, whereas tacrolimus binds to FK-binding proteins (FKBPs). The resulting complexes inhibit calcineurin activity, thereby preventing activation of the nuclear factor of activated T cells (NFAT) and suppressing T-cell activation and cytokine production [4]
	Tacrolimus TAC	Tacrolimus binds to FK-binding proteins (FKBPs), forming a complex that inhibits calcineurin activity and subsequently suppresses T-cell activation [5]
Antiproliferative agents	Mycophenolate Mofetil (MMF), Mycophenolate Sodium and Azathioprine	Mycophenolate mofetil, a prodrug of mycophenolic acid, inhibits inosine monophosphate dehydrogenase and suppresses lymphocyte proliferation, thereby reducing the risk of acute graft rejection [6,7]
Mammalian target of rapamycin inhibitors mTOR inhibitors	Sirolimus and Everolimus	mTOR inhibitors suppress T-cell activation and B-cell differentiation by blocking cytokine-mediated signal transduction and arresting cell-cycle progression from the G1 to the S phase [8]
Steroids	Prednisone	Corticosteroids exert potent anti-inflammatory and immunosuppressive effects. They inhibit the migration of neutrophils and monocytes, reduce the production of pro-inflammatory cytokines, including IL-1, IL-6, and TNF-α, and suppress the activity of T and B lymphocytes [9].
Conversion from CNIs	Belatacept	Belatacept is a selective T-cell costimulation blocker used as an alternative to calcineurin inhibitors in selected transplant recipients. Conversion from CNI-based therapy to belatacept-based therapy is an established strategy to reduce CNI-associated toxicity [10,11]

Calcineurin inhibitors (CNIs), including cyclosporine, voclosporin, tacrolimus, and pimecrolimus, exert potent immunosuppressive effects and are among the most widely used agents in maintenance immunosuppressive regimens [12]. Cyclosporine and tacrolimus are the best-established representatives of this drug class; however, their long-term use is associated with significant nephrotoxicity and may contribute to the development of chronic kidney injury.

The introduction of effective immunosuppressive therapy has markedly improved both graft and patient survival following transplantation. Nevertheless, accumulating evidence indicates that long-term exposure to calcineurin inhibitors (CNIs) may promote fibrotic remodeling in various tissues and organs. In the kidney, CNI-associated lesions include interstitial fibrosis, Bowman’s capsule fibrosis, and glomerulosclerosis, whereas fibrotic changes have also been reported in other organs [13]. They may also contribute to the development of hypertension and dyslipidaemia. Furthermore, calcineurin inhibitors have been implicated in direct cardiovascular toxicity, including the induction of cardiomyocyte apoptosis and the promotion of myocardial fibrosis [14]. In cases of intolerance to calcineurin inhibitors following kidney transplantation, conversion to belatacept-based immunosuppression may be considered. Belatacept is a non-nephrotoxic immunosuppressive agent that has emerged as an alternative to CNI-based therapy. A retrospective study by Divard et al. (2024), including 311 kidney transplant recipients treated between 2007 and 2020, demonstrated that conversion to belatacept was associated with a reduced risk of graft failure and superior long-term graft survival [11].

Mycophenolate mofetil (MMF), an immunosuppressive agent belonging to the class of antiproliferative drugs, is generally well tolerated by solid organ transplant recipients. However, its use may be associated with a range of adverse effects, including gastrointestinal and hematological complications, increased susceptibility to infections and malignancies, as well as potential teratogenicity [15,16].

Similarly to MMF, immunosuppressive agents belonging to the mTOR inhibitor class, such as everolimus, are generally well tolerated by solid organ transplant recipients [17]. However, mTOR inhibitors may also be associated with a variety of adverse effects. These include viral infections [18] and cardiovascular complications [19]. Glucocorticoids are also widely used as immunosuppressive agents in maintenance therapy following solid organ transplantation. However, long-term corticosteroid treatment may increase the risk of bacterial, fungal, and viral infections [20].

Animal models are frequently used to investigate the adverse effects of long-term immunosuppressive therapy on various organs and tissues. Previous studies have demonstrated the effects of prolonged multidrug immunosuppression on the salivary glands [21], kidneys [22], prostate gland [23] and other organs [24,25].

Following organ transplantation, immunosuppressive therapy typically involves the administration of several drugs in specific combinations. The choice of immunosuppressive regimen depends on the type of transplanted organ, the patient’s immunological risk, the severity of metabolic disorders, the presence of comorbidities, and graft function. Most transplant recipients receive a three-drug immunosuppressive regimen designed to balance efficacy in preventing graft rejection with safety by minimizing drug-related toxicity [26].

In the present study, we investigated the effects of long-term treatment with calcineurin inhibitor (CNI)-based triple immunosuppressive regimens containing cyclosporine A or tacrolimus in combination with rapamycin and a glucocorticoid on the development of collagen deposition in the jejunal mucosa of rats.

## 2. Materials and Methods

### 2.1. Research Material and Conducting an Experiment on Animals

The study was conducted using archival jejunal tissue specimens obtained from 14-week-old sexually mature male rats and preserved in paraffin blocks. The present study is a continuation of our previous investigation. The experimental protocol was approved by the Local Ethics Committee for Animal Experiments in Szczecin [27].

The animals were treated with the following immunosuppressive drugs at the doses presented in Table 2: (i) cyclosporine A (Sandimmun Neoral; C)—5 mg/kg body weight/day; (ii) tacrolimus (Prograf; T)—4 mg/kg body weight/day; (iii) rapamycin (Rapamune; R)—0.5 mg/kg body weight/day; and (iv) prednisone (Encorton; G)—4 mg/kg body weight/day. The doses of immunosuppressive drugs were selected based on previously established experimental protocols in rats. In particular, tacrolimus at a dose of 4 mg/kg/day has been used in previous studies investigating the effects of chronic immunosuppressive therapy in rat models [28,29]. Due to interspecies differences in pharmacokinetics and drug metabolism, doses expressed as mg/kg in rats should not be directly compared with those used clinically in human transplant recipients. Animals assigned to the experimental groups (CRG and TRG) received immunosuppressive treatment for six months according to calcineurin inhibitor (CNI)-based triple-drug regimens (Table 2).

### 2.2. Histological Staining

Intestinal tissue sections were stained with hematoxylin and eosin (H&E) for morphological evaluation. Collagen fibers within the jejunal mucosa were visualized using the Mallory trichrome staining method (Bio-Optica, Milan, Italy). In this staining procedure, collagen fibers were stained dark blue. Histological assessment of the examined tissues was performed using a light microscope (Leica DM5000B, Wetzlar, Germany).

### 2.3. Digital Image Analysis

To assess the content of collagen fibers, jejunal tissue sections stained with the Mallory trichrome method were subjected to quantitative digital image analysis using the PatternQuant 2.4 image analysis software (3DHISTECH Ltd., Budapest, Hungary). The following parameters were evaluated in the jejunal mucosa of both the control and experimental groups: the area, perimeter, and staining intensity of the analyzed collagen fibers. Multiple measurements were obtained from several microscopic fields for each animal. The measurements were expressed in pixels and subsequently used for statistical analysis. For each animal, the median value of each analyzed parameter was calculated from all measurements obtained from that animal. These individual animal-level median values were subsequently used for statistical analysis. The total number of measurements was 345 in the Control group, 176 in the CRG group, and 133 in the TRG group. The results are presented in Table 3.

### 2.4. Statistical Analysis

Multiple measurements of collagen fiber parameters were obtained from several microscopic fields for each animal using quantitative digital image analysis. A total of 345 measurements were obtained in the Control group, 176 measurements in the CRG group, and 133 measurements in the TRG group. To account for the repeated measurements obtained from the same animal and to avoid pseudoreplication, the median value for each analyzed parameter was calculated separately for each individual animal. These animal-level median values were subsequently used as the units of observation in the statistical analysis. Accordingly, the effective sample sizes were 6 animals in the Control group, 4 animals in the CRG group, and 6 animals in the TRG group.

For each animal, the median value of each analyzed parameter was first calculated from all measurements obtained from that animal. These individual animal-level median values were subsequently used for statistical analysis. The distribution of the variables was assessed using the Shapiro–Wilk test. Due to the small and unequal sample sizes of the study groups (Control, n = 6; CRG, n = 4; TRG, n = 6), non-parametric statistical methods were applied. Differences among the three groups were assessed using the Kruskal–Wallis test. When a statistically significant difference was detected, post-hoc pairwise comparisons were performed using Dunn’s test with Holm correction for multiple comparisons. A *p*-value < 0.05 was considered statistically significant.

## 3. Results

### 3.1. Morphology

**Figure 1:**
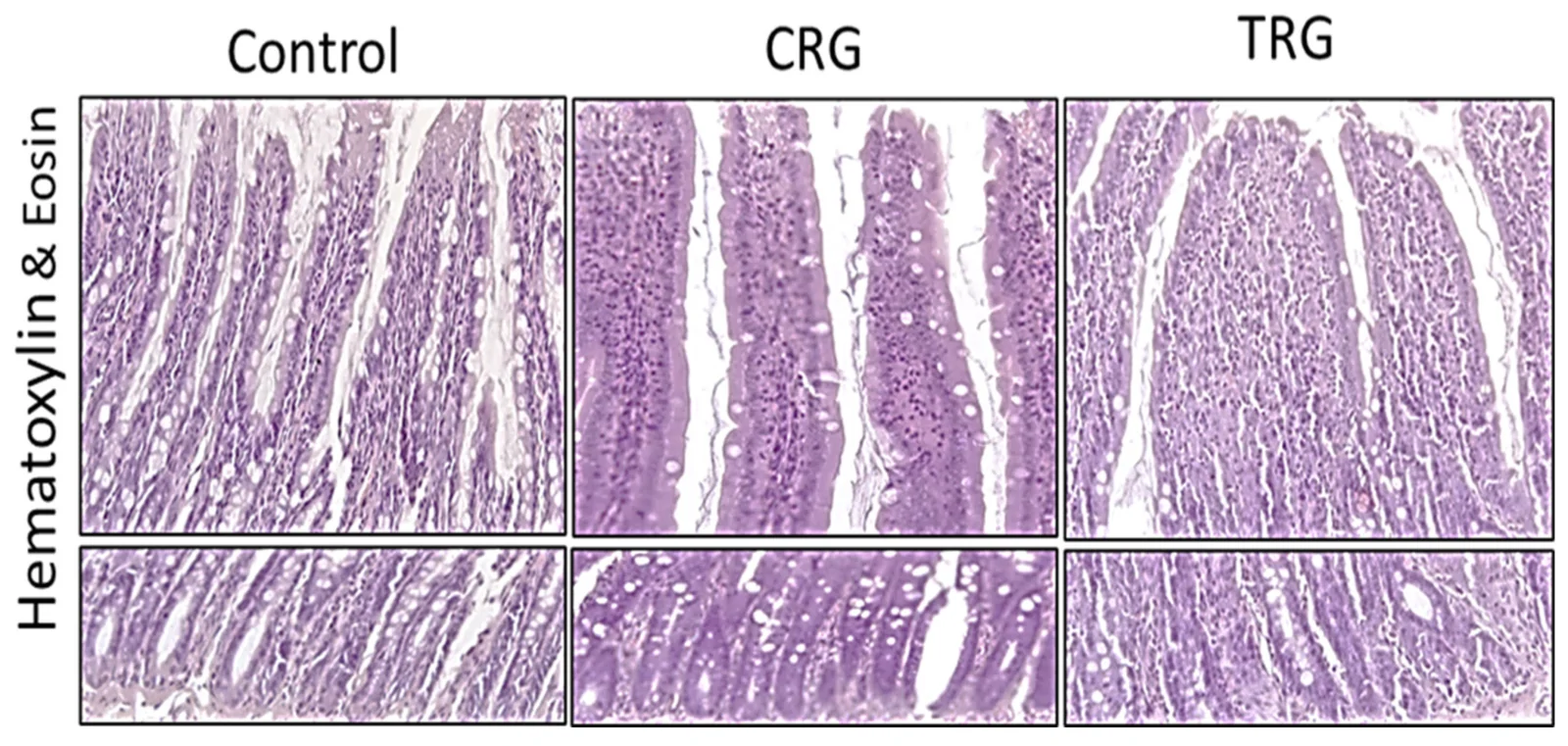
Morphology of the jejunal mucosa in rats from the control group and the two experimental groups receiving calcineurin inhibitor-based immunosuppressive regimens in combination with rapamycin and a glucocorticoid (CRG and TRG). Intestinal villi (upper part) and crypts (lower part) within the lamina propria of the mucosa are shown. Hematoxylin and eosin (H&E) staining; magnification 40×.

Stained sections of the jejunum obtained from animals in the control group and the two experimental groups were examined using light microscopy and subjected to digital image analysis. As described above, the Mallory trichrome staining method enabled the visualization of collagen fibers (Figure 1).

**Figure 2:**
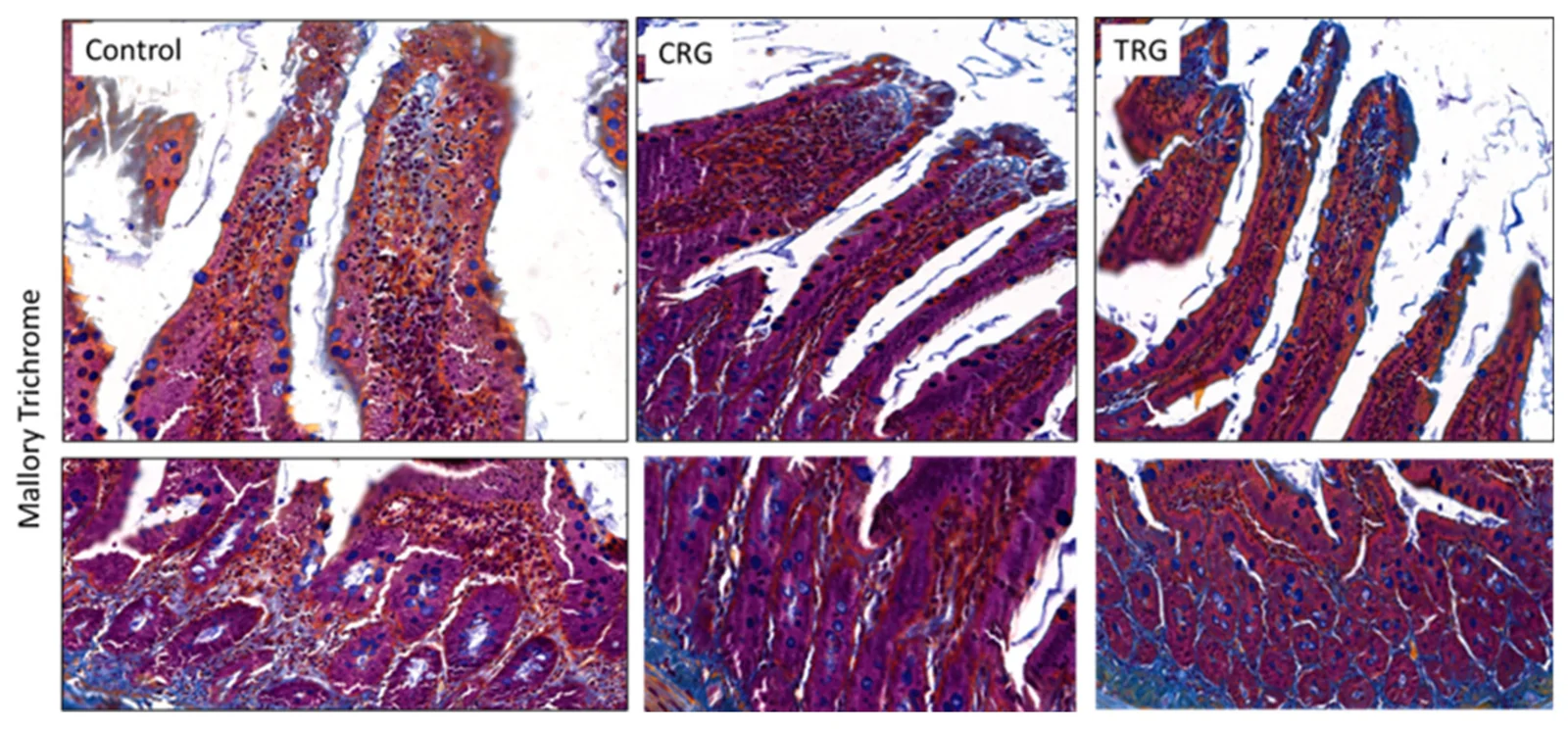
Jejunal mucosa of rats from the control group and the two experimental groups receiving calcineurin inhibitor-based immunosuppressive regimens (CRG and TRG). Intestinal villi and crypts within the lamina propria are shown. Collagen fibers within the mucosa are stained dark blue. Mallory trichrome staining; magnification 40×.

No apparent differences in the morphology of the jejunal mucosa were observed between the control and experimental groups. However, the jejunal mucosa of rats in the CRG group appeared more intensely stained than that of the control and TRG groups (Figure 2).

Qualitative histological assessment suggested increased collagen fiber deposition in the jejunal mucosa of both experimental groups compared with the control group.

### 3.2. Collagen Fiber Content—The Digital Analysis

**Figure 3:**
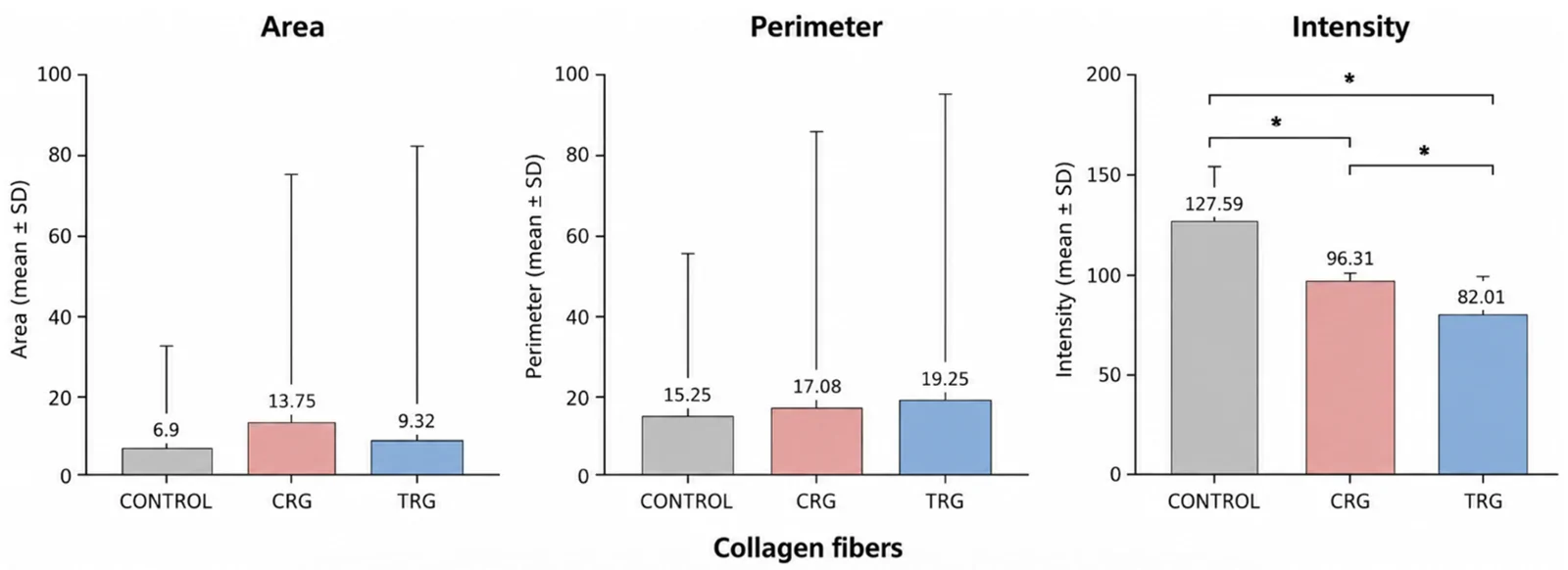
Quantitative digital image analysis of collagen fibers in the jejunal mucosa of rats from the control group (C) and the experimental groups receiving calcineurin inhibitor-based immunosuppressive regimens (CRG and TRG). Statistically significant differences between groups are indicated by dashed lines with a star (* *p* < 0.05).

Digital image analysis demonstrated differences in the content of collagen fibers among the examined groups. The highest mean area occupied by collagen fibers was observed in the CRG group (13.75 ± 61.80), followed by the TRG group (9.32 ± 72.88) and the Control group (6.90 ± 26.24) (Table 3, Figure 3).

The highest mean perimeter values of collagen fiber deposits were observed in the TRG group (19.25 ± 76.57), followed by the CRG group (17.08 ± 68.88) and the Control group (15.25 ± 39.33) (Table 3, Figure 3).

Analysis of mean staining intensity revealed the strongest staining reaction in the Control group (127.59 ± 25.62), followed by the CRG group (96.31 ± 6.71) and the TRG group (82.01 ± 13.38) (Table 3, Figure 3).

Statistical analysis using the Kruskal–Wallis test followed by Dunn’s post-hoc test with Holm correction demonstrated statistically significant differences in staining intensity among the examined groups. Pairwise comparisons revealed significant differences between the Control and CRG groups, between the Control and TRG groups, and between the CRG and TRG groups (Figure 3, Table 3).

## 4. Discussion

In general, the wall structure of the duodenum, jejunum, and ileum is similar throughout the length of the small intestine. It consists of three main layers: the mucosa, submucosa, and muscularis externa. The greatest morphological variation occurs within the mucosa.

The jejunal mucosa is composed of an epithelial layer, the lamina propria containing intestinal glands (crypts of Lieberkühn), and the muscularis mucosae. The lamina propria consists of loose connective tissue rich in fibroblasts and extracellular matrix components, including collagen, elastic, and reticular fibers, as well as ground substance. It also contains numerous elements of the gut-associated lymphoid tissue (GALT) [30]. It forms the core of the intestinal villi and fills the spaces between the crypts.

As mentioned above, the present study investigated the effects of long-term administration of calcineurin inhibitor-based immunosuppressive regimens on fibrotic remodeling of the jejunal mucosa in rats. Fibrosis is defined as a pathological process characterized by excessive accumulation of extracellular matrix components within tissues [31] (Siani et al. 2020). Progressive extracellular matrix deposition may disrupt normal tissue architecture, impair organ function, and ultimately contribute to organ failure [31]. It occurs when there is an excessive accumulation of collagen fibers—particularly types I and III—in the extracellular matrix of tissue [32].

A detailed analysis of our findings demonstrated that the cyclosporine-based triple immunosuppressive regimen was associated with greater collagen deposition in the jejunal mucosa than the tacrolimus-based regimen. However, because both experimental groups also received rapamycin and prednisone, the present experimental design does not allow the observed effects to be attributed exclusively to the individual calcineurin inhibitors. The area occupied by collagen fibers increased in the following order: control group < TRG < CRG, corresponding to values of 16.54%, 32.13%, and 44.71%, respectively. These findings are consistent with previous experimental studies demonstrating that tacrolimus possesses lower fibrogenic potential than cyclosporine under both in vivo and in vitro conditions [33,34,35].

Nevertheless, evidence from the prospective, open-label, 12-month REFINE study, which included 356 recipients of de novo liver transplants performed for hepatitis C virus-related cirrhosis, does not fully support these observations. In that study, no significant differences in fibrosis progression were observed between cyclosporine A- and tacrolimus-based regimens when administered in combination with corticosteroids. Interestingly, patients receiving cyclosporine A without corticosteroids exhibited reduced fibrosis progression during the first year following transplantation [35].

Retrospective studies conducted by Demirci et al. (2025) demonstrated the nephrotoxic effects of calcineurin inhibitors in kidney transplant recipients chronically exposed to cyclosporine A (CsA) and tacrolimus.

One of the adverse consequences of long-term CNI therapy was the development of renal interstitial fibrosis. Myofibroblasts located in the vicinity of injured renal tubules and glomeruli, as well as pericytes surrounding blood vessels, exhibited profibrotic activity and contributed to extracellular matrix accumulation. Notably, these fibrotic processes were more pronounced in animals receiving cyclosporine than in those treated with tacrolimus [36]. These findings are consistent with the results of the present study, which demonstrated more pronounced collagen deposition in the jejunal mucosa of rats receiving the cyclosporine-based regimen.

The connective tissue of the mucosa in the small intestine contains mesenchymal cells that contribute to fibrosis. These include fibroblasts, myofibroblasts, pericytes, stem cells, and smooth muscle cells. All of these cells can produce extracellular matrix (ECM) components and participate in their turnover [37]. Extracellular matrix (ECM) turnover is tightly regulated by matrix metalloproteinases (MMPs) and their endogenous inhibitors, tissue inhibitors of metalloproteinases (TIMPs). The balance between ECM synthesis and degradation is essential for maintaining tissue homeostasis, preserving intestinal architecture, and supporting normal intestinal motility. Disruption of this balance may lead to excessive ECM accumulation and the development of fibrosis [38,39]. The presence of profibrotic cells within the small intestinal mucosa may promote an imbalance between matrix metalloproteinases (MMPs) and their inhibitors (TIMPs) in the connective tissue of organ transplant recipients receiving calcineurin inhibitor-based maintenance immunosuppression. However, these potential mechanisms were not directly investigated in the present study and should therefore be regarded as possible biological explanations for the observed collagen accumulation rather than mechanisms demonstrated by the current findings.

In our previous study, long-term administration of calcineurin inhibitor-based triple-drug regimens (CMG and TMG) disrupted extracellular matrix (ECM) homeostasis in the jejunal wall of rats. Specifically, the MMP-2/TIMP-2 balance was disturbed, with more pronounced alterations observed in the tacrolimus-treated group. These findings suggest that prolonged immunosuppressive therapy may affect extracellular matrix remodeling, thereby contributing to the development of fibrotic changes observed in the present study [40].

The present study has several limitations. First, the relatively small and unequal group sizes, particularly the reduced number of animals in the CRG group, may limit the statistical power of the study. Second, because both experimental groups received multidrug immunosuppressive regimens, the observed effects cannot be attributed exclusively to individual calcineurin inhibitors. Third, the assessment of extracellular matrix remodeling was based primarily on histological visualization and quantitative analysis of collagen fibers. Therefore, although the observed increase in collagen deposition is consistent with fibrotic remodeling, additional molecular and cellular markers would be required to comprehensively characterize the fibrotic process. Finally, potential mechanisms involving fibroblast or myofibroblast activation and regulation of extracellular matrix turnover were not directly investigated in the present study and should be interpreted in the context of previously published evidence.

## 5. Conclusions

The findings of the present study may differ from those reported by other investigators. However, it should be emphasized that our experimental model was based on long-term administration of clinically relevant triple-drug immunosuppressive regimens containing calcineurin inhibitors, which remain the cornerstone of maintenance therapy following solid organ transplantation. This approach distinguishes our study from many previous experimental investigations, which have predominantly evaluated the effects of single immunosuppressive agents. Therefore, our findings may more closely reflect the conditions encountered in routine clinical practice.

## Figures and Tables

**Table 2:** Triple-drug immunosuppressive regimens administered to rats during the experiment and the number of animals (*n*) included in each experimental group. During the study, two animals from the CRG group died; therefore, the final number of animals in this group was four.

Group 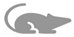	(Sandimmun-Neoral) Cyclosporin A (C) 5 mg/kg Body Weight/Day	(Rapamune) Rapamycin (R) 0.5 mg/kg Body Weight/Day	(Prograf) Tacrolimus (T) 4 mg/kg Body Weight/Day	(Encorton) Prednisone Glucocorticosteroid (G) 4 mg/kg Body Weight/Day
Control n = 6	−	−	−	−
Group CRG n = 6/4	+	+	−	+
Group TRG n = 6	−	+	+	+

“+” indicates that the drug was included in the regimen; “−” indicates that the drug was not included.

**Table 3:** Statistical analysis of the results, obtained from the quantitative digital evaluation of the studied tissues (* *p* < 0.05).

Collagen Fibers	Control	CRG	TRG
Area			
Number of measurements	345	176	133
n (animals)	6	4	6
Mean ± SD	6.9 ± 26.24	13.75 ± 61.80	9.32 ± 72.88
Median	0.33	0.21	0.15
Perimeter			
Number of measurements	345	176	133
n (animals)	6	4	6
Mean ± SD	15.25 ± 39.33	17.08 ± 68.88	19.25 ± 76.57
Median	3.18	2.72	2.55
Intensity			
Number of measurements	345	176	133
n (animals)	6	4	6
Mean ± SD	127.59 ± 25.62	96.31 ± 6.71 *	82.01 ± 13.38 *
Median	131	96	84

## Data Availability

The data presented in this study are available from the corresponding author upon reasonable request.

## Abbreviations

The following abbreviations are used in this manuscript:

CNIs	Calcineurin Inhibitors
CyA	Cyclosporine A
MMF	Mycophenolate Mofetil
mTOR inhibitors	Mammalian target of rapamycin inhibitors
TAC	Tacrolimus
